# Extracellular Vesicular Proteins in Plasma from Patients with Cutaneous Lupus Correlate with Disease Activity

**DOI:** 10.3390/cimb48010013

**Published:** 2025-12-23

**Authors:** Mariko Ogawa-Momohara, Avital Baniel, Nilesh Kodali, Fazelinia Hossein, Hua Ding, Spruce Lynn, Julianne Kleitsch, DeAnna Diaz, Thomas Vazquez, Victoria P. Werth

**Affiliations:** 1 Corporal Michael J. Crescenz Veterans Affairs Medical Center, Philadelphia, PA 19104, USA; marikkori0910@gmail.com (M.O.-M.);; 2 Department of Dermatology, School of Medicine, University of Pennsylvania, Philadelphia, PA 19104, USA; 3 Department of Dermatology, Nagoya University Graduate School of Medicine, Nagoya 466-8550, Japan; 4 Proteomics Core, The Children’s Hospital of Philadelphia, Philadelphia, PA 19104, USA; 5 Department of Dermatology, Perelman Center for Advanced Medicine, University of Pennsylvania, Suite 1-330A, 3400 Civic Center Boulevard, Philadelphia, PA 19104, USA

**Keywords:** Cutaneous lupus erythematosus (CLE), extracellular vesicles (EV), CLASI, SLE, SLEDAI

## Abstract

Cutaneous lupus erythematosus (CLE) can occur independently of lupus erythematosus. SLE, and its responsiveness to treatment, does not necessarily align with that of coexisting SLE. Extracellular vesicles (EVs) allow communication between cells and rapid delivery throughout the body. We hypothesized that EVs may support disease-specific inflammation in CLE and SLE patients. Plasma EVs from healthy controls (n = 5), CLE (n = 6), and dermatomyositis (n = 17) were purified by ultracentrifugation and size-exclusion chromatography, phenotyped by flow cytometry, and profiled by LC-MS/MS. Circulating EVs were mainly platelet-, endothelial-, and antigen-presenting cell-derived examples. CLE EVs harbored four proteins absent in the controls—mimecan, IFI27, fibulin-2, and snRNP B/B′ (anti-Sm an-tigens)—and their cumulative number increased with SLEDAI. Relative to the controls, 18 proteins were upregulated and 15 downregulated in CLE EVs. The number of upregulated proteins showed a trend toward a correlation with SLEDAI (r = 0.79, *p* = 0.06) but not with CLASI (r = 0.21). Among upregulated proteins, lysozyme C and hyaluronan-binding protein 2 tracked with cutaneous activity (CLASI r = 0.74 and r = 0.86) but not with systemic activity (SLEDAI r = 0.52 and r = 0.31). CLE plasma EVs were enriched in antigen-presenting cell markers and disease-related cargo, including anti-Sm antigens and proinflammatory proteins. Although overall protein diversity correlated primarily with systemic disease activity, a subset of proteins appeared to reflect cutaneous activity.

## 1. Introduction

SLE is a chronic multifactorial autoimmune connective tissue disease characterized by autoantibody production, inflammation, and tissue damage in multiple organs resulting from the activation of several proinflammatory pathways [1,2]. SLE is a highly heterogeneous disease. Many patients are refractory to multiple immunosuppressive agents, highlighting the need for new treatments and targeted therapies [3]. The skin is one of the most commonly affected organs in SLE patients. Cutaneous lupus erythematosus (CLE) has similar immune pathways activated as in SLE, but the clinical manifestations of CLE may occur with or without SLE [4,5]. Therefore, new markers are needed to better classify CLE and assess disease activity.

Extracellular vesicles (EVs) are small, lipid bilayer membrane structures that are actively released by normal, diseased cells and are widely distributed by almost all cell types of the human body in vitro and in vivo. EVs can carry a variety of molecules, such as nucleic acids (DNA, long non-coding RNA, microRNA, mRNA, tRNA-derived small RNA), lipids and proteins (channels, adhesion molecules, receptors, cytokines), to mediate cell–cell communication [6]. EVs are ubiquitous in body fluids, such as cerebrospinal fluid, saliva, milk, urine, blood, and semen, and reach all parts of the body through the bloodstream. EVs have low antigenicity and have specific migratory properties, making them a potential mechanism for drug delivery [7].

Recent advances in EV biology and methodology have established standardized approaches for EV isolation and characterization. International guidelines and technical reviews describe the best practices for size-exclusion chromatography (SEC), ultracentrifugation (UC), and density gradient and sedimentation velocity (SV)–based methods, enabling more rigorous isolation of EV subtypes [8,9].

Many of the molecules carried by EVs activate intrinsic immunity. We previously reported that dermatomyositis (DM) plasma contains a higher number of EVs than healthy controls and that EVs isolated from DM plasma triggers proinflammatory cytokines (IL-6, TNFa), including type I interferon (IFN-I) release via the STING signaling pathway [10]. The proinflammatory properties of EVs in the blood of patients with autoimmune diseases have been demonstrated by others as well. Kato et al. reported that EVs in SLE serum promote IFN-I production and interferon-stimulated gene (ISG) induction through the cGAS–STING pathway [11]. Recent studies have demonstrated that circulating EV cargo—ranging from microRNA and lncRNA to inflammatory proteins—correlates with disease activity in autoimmune conditions, including SLE, and may contribute to systemic inflammatory pathways [12,13].

Several studies have shown that microRNAs and tRNA-derived small RNAs (tsRNAs) contained in SLE blood EVs correlate with SLE disease activity. Proteins in EVs produced by T cells and macrophages isolated from the blood of SLE patients have been reported to have proinflammatory effects [14]. These results indicate that the amount of EVs in the blood of patients with inflammatory diseases is increased, and that there is an inflammation-inducing action. We hypothesized that specific pro-inflammatory EV proteins are elevated in the blood of CLE patients.

The primary objective of this study was to characterize pro-inflammatory EV proteins in the blood of patients with CLE.

## 2. Materials and Methods

### 2.1. Patient Enrollment

A total of 28 subjects, 6 CLE, 17 DM patients, and 5 healthy controls (HCs), were recruited from the Department of Dermatology at the Hospital of the University of Pennsylvania (Philadelphia, PA, USA) with appropriate Institutional Review Board approval. All subjects signed a written informed consent form prior to participation in this study. Patients were diagnosed with CLE according to the Gilliam classification. SLE was diagnosed according to the 1997 American College of Rheumatology classification criteria. SLE disease activity was assessed using the Systemic Lupus Erythematosus Disease Activity Index 2000 (SLEDAI-2K) score. Skin disease activity was determined using the Cutaneous Lupus Erythematosus Disease Area and Severity Index (CLASI). DM was diagnosed using Bohan and Peter criteria, Sontheimer criteria, or investigator/expert experience. CLASI and SLEDAI were recorded at the time of blood sampling. DM samples were included as an autoimmune disease control to allow disease-specific filtering of EV proteins.

### 2.2. Isolation and Purification of Extracellular Vesicles

**Figure A1:**
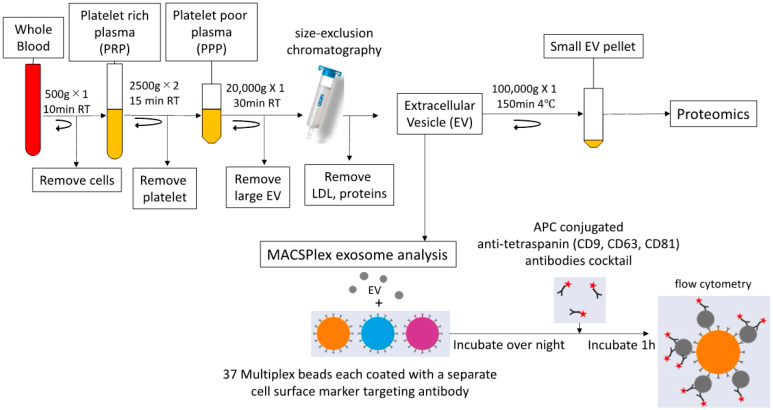
Experimental diagram.

Heparinized venous blood from CLE, DM patients, or HCs was centrifuged at 500× *g* for 10 min to obtain cell-free plasma. Within hours of blood collection, the cell-free plasma was centrifuged twice at 2500× *g* for 15 min to remove debris and platelets. For proteomic analysis, 4 mL of the platelet-poor plasma was centrifuged at 20,000× *g* for 30 min to remove large EVs (lEVs). The collected lEV-depleted supernatants were then applied to a size exclusion chromatography column (qEV2, Izon Science, Christchurch, New Zealand) to isolate EVs, centrifuged at 100,000× *g* for 150 min at 4 °C to pellet small extracellular vesicles (sEVs), and stored at −80 °C within 24 h after blood collection. For MACSplexs analysis, 500 μL of lEV-depleted plasma was applied to a size exclusion chromatography column (qEV original, Izon Science) (Figure A1).

### 2.3. Extracellular Vesicle Validation

To verify the presence and purity of extracellular vesicles (EVs) in accordance with the MISEV2018 guidelines, we performed three complementary validation analyses.

Nanoparticle tracking analysis (NTA): Particle concentration and size distribution were measured using a Zetaview TWIN (Particle Metrix GmbH, Meerbusch, Germany). EV samples were diluted in phosphate-buffered saline (PBS).

Western blotting for EV markers: EVs isolated from 4 mL of plasma obtained from 5 healthy controls were concentrated to 100 µL using a centrifugal ultrafiltration filter (Merck Milipore Ltd., Carrigtwohill, Ireland). Samples were dissolved in a 2× sample buffer, separated on 4–15% SDS-PAGE, and transferred to a polyvinylidene fluoride membrane. After overnight blocking at 4 °C with skim milk, membranes were incubated for 1 h at room temperature with an anti-CD81 antibody (sc-166029, Santa Cruz, Dallas, TX, USA; 1:100 dilution), a canonical tetraspanin EV marker. To assess contamination from abundant plasma proteins and intracellular organelle components, additional Western blotting was performed using antibodies against albumin and calnexin. HaCaT keratinocyte lysates were used as a positive control for intracellular protein contamination. For albumin detection, membranes were incubated with an anti-albumin antibody (ProteintechGroup, Inc., Chicago, IL, USA, 16475-1-AP; 1:5000). Calnexin, an endoplasmic reticulum marker, was evaluated using an anti-calnexin antibody (Cell Signaling Technology, Danvers, MA, USA, #2679; 1:1000). After incubation with HRP-conjugated secondary antibodies, signals were visualized by chemiluminescence. Albumin was detectable in whole plasma but not in EV preparations, whereas calnexin was detected only in HaCaT cell lysates and absent from EVs, confirming minimal contamination and supporting EV purity.

Transmission electron microscopy (TEM): Concentrated EVs were diluted in PBS, applied to carbon-coated grids (EM-JAPAN, Tokyo, Japan), negatively stained with uranyl acetate, and imaged using a JEM-1400PLUS transmission electron microscope (JEOL Ltd., Tokyo, Japan).

### 2.4. Multiplex Exosome Flow Cytometry Assay

To identify the major source of EVs in plasma, a bead-based multiplex exosome flow cytometry assay using a MACSPlex Exosome Kit (human; Miltenyi Biotec GmbH, Bergisch Gladbach, Germany) was tested. EV samples at a 10 μg protein concentration were incubated overnight with multiplexed beads coated with 37 different cell surface markers, including CD3, CD4, CD19, CD8, HLA-DR/DP/DQ, CD56, CD105, CD2, CD1c, CD25, CD49e, ROR1, CD209, CD9, SSEA-4, HLA-A/B/C, CD63, CD40, CD62P, CD11c, CD81, MCSP, CD146, CD41b, CD42a, CD24, CD86, CD44, CD326, CD133/1, CD29, CD69, CD142, CD45, CD31, CD20, and CD14 and two isotype controls (mIgG1 and REA). The tetraspanin-conjugated APC signal (conjugated with tetraspanin CD9, CD63, CD81) was measured by flow cytometry. Data were acquired (>1 × 10^4^ events) on a BD LSRII 4-laser flow cytometer (BD Biosciences, San Jose, CA, USA) and analyzed using FlowJo^®^ software (version 10.8.1; BD Life Sciences, Ashland, OR, USA).

### 2.5. Mass Spectrometry

#### 2.5.1. Digestion of EVs

EVs were lysed, solubilized, and digested on an S-Trap (Protifi, LLC, Fairport, NY, USA) as per the manufacturer’s protocol [15]. The resulting peptides were desalted using an Oasis HLB plate (Waters Corporation, Milford, MA, USA), dried by vacuum centrifugation, and reconstituted in 0.1% Trifluoroacetic Acid (TFA) containing iRT peptides (Biognosys AG, Schlieren, Switzerland).

#### 2.5.2. Mass Spectrometry Acquisition

Peptides were analyzed on an Exploris 480 mass spectrometer (ThermoFisher Scientific, San Jose, CA, USA) coupled with an Ultimate 3000 nano-UPLC system and an EasySpray source using data independent acquisition (DIA). Raw mass spectrometry data were searched using the direct-DIA mode in Spectronaut 15 software (Biognosys AG, Schlieren, Switzerland) [16]. The protein MS2 intensity values generated by Spectronaut were utilized for bioinformatics analysis.

Please refer to the Supplementary Materials for detailed information on the mass spectrometry experimental procedures.

### 2.6. Statistical Analysis

The following statistical methods refer to all analyses except for the mass spectrometry-based proteomic analysis, for which dedicated procedures are described below. All statistical analyses were performed using GraphPad Prism (version 10.0; GraphPad Software, San Diego, CA, USA). Data were first assessed for normality using the Shapiro–Wilk test. For normally distributed data, group comparisons were performed using unpaired two-tailed Student’s *t*-tests (two groups) or one-way ANOVA followed by Tukey’s post hoc test (three or more groups). For non-normally distributed data, the Mann–Whitney U test (two groups) or Kruskal–Wallis test with Dunn’s correction (three or more groups) was applied. Correlation analyses between EV proteins and clinical parameters (e.g., CLASI, SLEDAI) were conducted using Spearman’s rank correlation coefficient. All statistical tests were two-sided unless otherwise specified. A *p*-value < 0.05 was considered statistically significant.

## 3. Results

### 3.1. Patient Demographics

The mean age of the 6 CLE patients was 61.5 years (median 65.5 years), 100% (6/6) were female, 50% (3/6) were Caucasian, and 50% (3/6) were African American. A total of 67% (4/6) of the CLE patients were diagnosed with SLE (Table 1). For disease control, 17 DM patients were included. The mean age of the DM patients was 59.3 years (median 59 years); 70% (12/17) were female. Amyopathic DM was dominant in the 64.7% cohort (11/17) and the rest were classic DM 35% (6/17). Two DM patients had cancer. A total of 60% (3/5) were female in the healthy controls (HCs) (Supplementary Table S2).

### 3.2. Extracellular Vesicle Validation

The diameters of the recovered EVs measured using nanoparticle tracking analysis (NTA) revealed a sharp peak at approximately 100 nm. This confirmed the collection of uniformly small EVs (Supplementary Figure S1A). Transmission electron microscopy was used to observe the morphology of the extracted EVs, demonstrating that they were formed by a lipid bilayer with minimal contamination from aggregated proteins (Supplementary Figure S1B). Additionally, Western blot analysis confirmed the presence of CD81, a tetraspanin known to be located on the surface of exosomes, in the extracted EVs (Supplementary Figure S1C). Furthermore, to evaluate potential contamination from intracellular components or abundant plasma proteins, we performed Western blotting for negative markers. Calnexin, an endoplasmic reticulum protein and a standard indicator of cellular contamination, was readily detected in HaCaT cell lysates but was completely absent in the EV fractions. Conversely, albumin, one of the most abundant soluble proteins in plasma, was present in whole plasma but undetectable in the isolated EVs. These findings confirm that the EV preparations were largely free of intracellular debris and soluble plasma protein contamination, supporting the successful and specific isolation of small EVs (Supplementary Figure S1D).

### 3.3. Identification of Major EV-Producing Cells in Plasma by Multiplex Exosome Flow Cytometry

**Figure 1:**
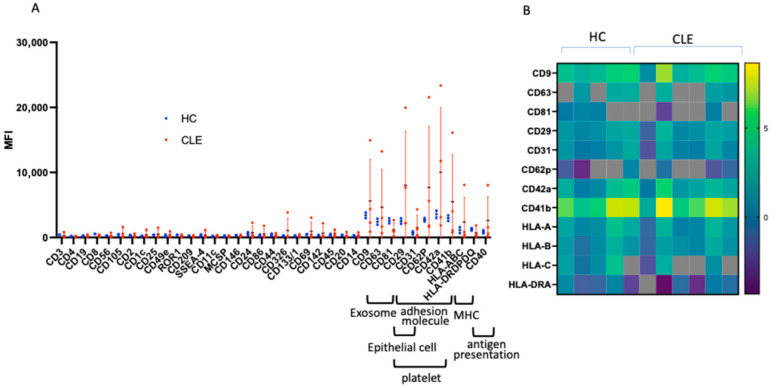
Major EV surface markers and protein expression in proteomics. (**A**) MACSPlex bead flow cytometry revealed that 11 out of 37 types of lymphocytes and cell surface markers were elevated in EVs from HC and CLE patients. Blood EVs were mainly derived from platelets, endothelial cells, and antigen-presenting cells. (**B**) Among the 37 surface markers, the expression of 10 markers other than CD40 was also confirmed by proteomic analysis. Heatmap colors correspond to log2-transformed MS2 intensity values.

EVs collected from the plasma of 4 CLE patients and 2 HCs were included in the proteomics analysis to identify the major source of EVs in plasma. Among 37 cell surface markers, 11 markers were upregulated. Their mean fluorescence intensity (MFI) was more than twice that of the negative controls (mIgG1 and REA) in each sample. The 11 upregulated markers included 3 tetraspanin markers (CD9, CD63, CD81), 5 adhesion molecules (CD29, CD31, CD62P, CD42a, CD41b), MHC class I (HLA-A/B/C), MHC class II (HLA-DR/DP/DQ), and CD40. CD31 and CD62P are mainly expressed on epithelial cells; CD62P, CD42a, and CD41b are platelet markers; and MHC class II and CD40 are both important molecules for antigen presentation. From these results, we concluded that the major source of EVs in plasma is epithelial cells, platelets, and antigen-presenting cells (Figure 1A).

We also examined the expression of 37 cell surface markers. In general, proteomics analysis is less sensitive than methods such as flow cytometry that identify single markers. Of the 37 markers, 10 proteins were identified by proteomics analysis, and of the 11 markers shown to be increased by flow cytometry, expression was confirmed for all except CD40. This consistency indicates the accuracy of the proteomics analysis (Figure 1B).

### 3.4. Identification of CLE Cohort Specific Proteins Using Proteomics Analysis

**Figure 2:**
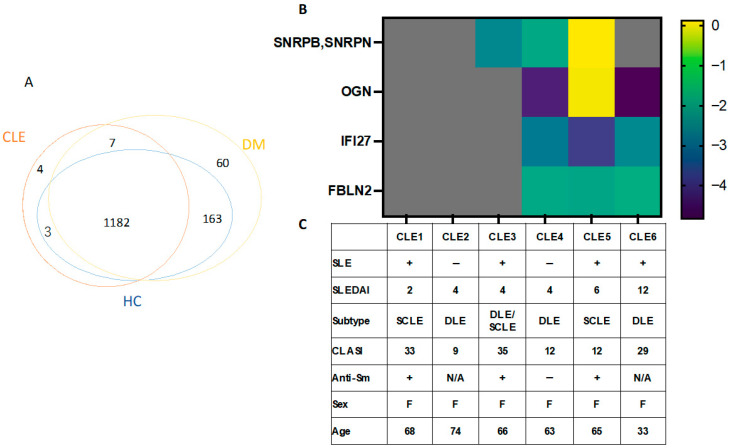
LE specific proteins in EV. (**A**) Proteomic analysis revealed 4 CLE cohort specific proteins (red overlay). The Venn diagram shows the number of EV proteins differentially identified in HCs, CLE, and DM patients. (**B**) Heatmap of the protein expression of 4 CLE cohort specific proteins. The gray areas indicate that no protein was found. Heatmap colors correspond to log2-transformed MS2 intensity values. (**C**) Clinical plots of 6 CLE patients. CLASI = cutaneous lupus erythematosus area and severity index, DLE = discoid lupus erythematosus, SCLE = subacute cutaneous lupus erythematosus, SLE = systemic lupus erythematosus, SLEDAI = SLE disease activity index.

DM served as a disease control to exclude proteins shared with DM and to determine CLE-specific EV cargo. Proteomic analysis identified an average of 975.9 proteins in DM, 1041.1 in CLE, and 1052.8 in HCs. There was no significant difference in the number of proteins between the three groups (*p* = 0.8). A total of 1419 proteins were identified. Of these there were 4 CLE cohort specific proteins, 67 DM-specific proteins, and 7 proteins in both CLE and DM patients but not HCs. HCs had no unique proteins (Figure 2A). The four unique CLE proteins were SNRPB/SNRPN (small nuclear ribonucleoprotein-associated proteins B and B’/small nuclear ribonucleoprotein-associated protein N), OGN (mimecan), IFI27 (interferon alpha-inducible protein 27), and FBLN2 (fibulin-2). Protein expressions of OGN, IFI27, and FBLN2 were detected only in higher-score SLEDAI patients (CLE5, CLE6), but not in those with lower SLEDAI scores (CLE1, CLE2, CLE3) (Figure 2B,C). Small nuclear ribonucleoprotein-associated proteins B and B’ and small nuclear ribonucleoprotein-associated protein N are also known as Sm-B/B’ and Sm-D, both part of anti-Sm antigens. SNRPB and SNRPN proteins were detected in 3/6 CLE patients, and 2 were positive for anti-Sm antibodies (Figure 2B,C).

### 3.5. Protein Expression Differences Between CLE and HC Patients

**Figure 3:**
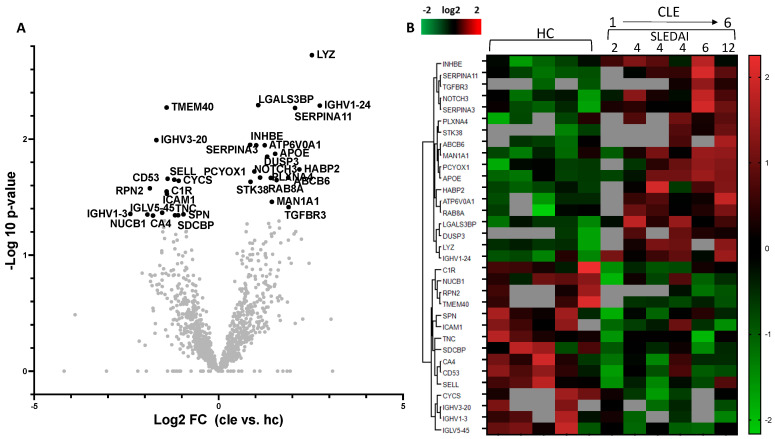
Thirty-three proteins with significant differences in expression levels between LE and HC patients. (**A**) Comparison of protein expression loaded on CLE plasma EVs vs. HC plasma EVs. Volcano plot of statistical significance against fold change between HC and CLE patients. Thirty-three differentially expressed proteins were identified. Twenty-nine proteins demonstrated |log2FC| > 1. (**B**) Heatmap for the 33 differentially expressed proteins between HC and CLE patients. The number of proteins whose expression level was increased in CLE patients correlated with the SLEDAI score.

Downstream quantitative comparisons were restricted to CLE versus HC patients because these addressed the primary study aim. A total of 1185 proteins detected in both CLE and HC patients were quantified. Figure 3A shows a volcano plot of the most frequent 1185 proteins. The 29 proteins whose expression was significantly increased or decreased (*p* < 0.05) and |log2FC| > 1 are highlighted. The expression levels of 33 molecules identified in the CLE group relative to the HC group are shown in a heatmap (Figure 3B). Among the 33 significantly differentially expressed proteins, 18 were upregulated and 15 were downregulated in CLE patients. CLE patients 1 through 6 are arranged from left to right in increasing order of SLEDAI values. The detailed list of up- and downregulated protein expressions between CLE and HC patients is shown in Supplementary Table S1. Because non-detection in mass spectrometry cannot distinguish between low abundance, true absence, or technical variation, proteins not detected in a given sample were treated as missing values and omitted from the correlation analysis.

**Figure 4:**
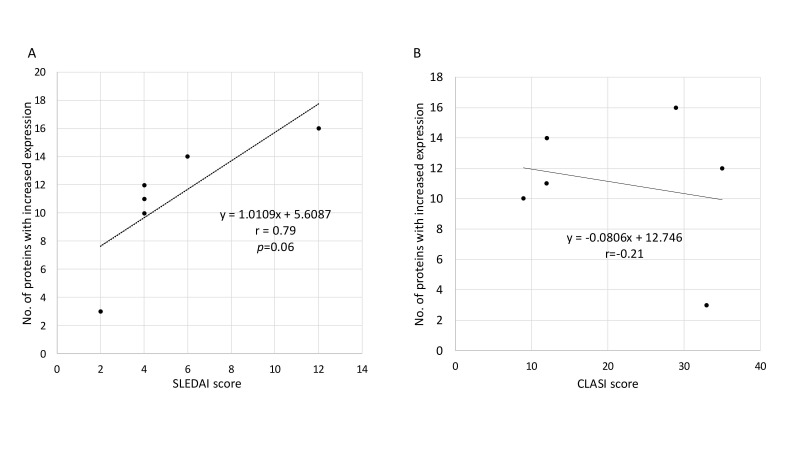
Correlation between SLEDAI, CLASI, and the number of elevated proteins in CLE plasma EVs compared to the healthy group. Samples without detectable protein levels were considered missing values and excluded from the correlation calculations. Correlation between disease activity indices and the number of elevated proteins in CLE plasma EVs compared to the healthy group. (**A**) Correlation between the Systemic Lupus Erythematosus Disease Activity Index (SLEDAI) score and the number of elevated proteins in CLE plasma EVs, showing a positive correlation (r = 0.79, *p* = 0.06). (**B**) Correlation between the Cutaneous Lupus Erythematosus Disease Area and Severity Index (CLASI) score and the number of elevated proteins in CLE plasma EVs, showing no significant correlation (r = −0.21).Samples without detectable protein levels were considered missing values and excluded from the correlation.

**Figure 5:**
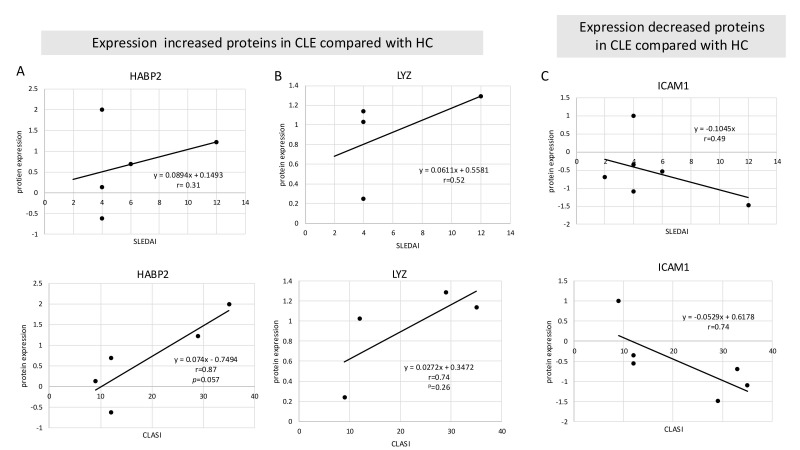
Plasma EV proteins showed a correlation with CLASI scores and SLEDAI. (**A**) Correlations between expression levels of HABP2, a protein that was increased in CLE EVs, and SLEDAI and CLASI scores. (**B**) Correlations between the expression levels of LYZ, a protein that was increased in CLE EVs, and SLEDAI and CLASI scores. (**C**) Correlations between expression levels of ICAM1, a protein that was decreased in CLE EVs, and SLEDAI and CLASI scores. Samples without detectable protein levels were considered missing values and excluded from the correlation calculations.

Among the 18 proteins with increased expression in CLE patients, the number of proteins that increased in each patient correlated with SLEDAI (r = 0.79) (Figure 4A), but not with CLASI (r = −0.21) (Figure 4B). However, several individual protein expression levels correlated better with CLASI than with SLEDAI (Figure 5A–C). HABP2 (hyaluronan-binding protein 2) is activated and increased during inflammation and is associated with coagulation. It is also known as factor VII activating protease (FSAP) and has been reported to be a protein necessary for the removal of extracellular chromatin [17], which can become an autoantigen in SLE. It showed a positive correlation with CLASI but no correlation with SLEDAI (Figure 5A). LYZ (lysozyme C), which was most significantly upregulated in CLE compared to HC patients (Figure 3A), is an antimicrobial response factor and had a positive correlation trend with CLASI (Figure 5B). Interestingly, ICAM1 (Intercellular Adhesion Molecule 1), which is a common adhesion molecule on endothelial cells and thought to be upregulated during inflammation, was downregulated in CLE patients and had a negative correlation with CLASI (Figure 5C).

### 3.6. Pathway Analysis of CLE EV Proteins

**Figure 6:**
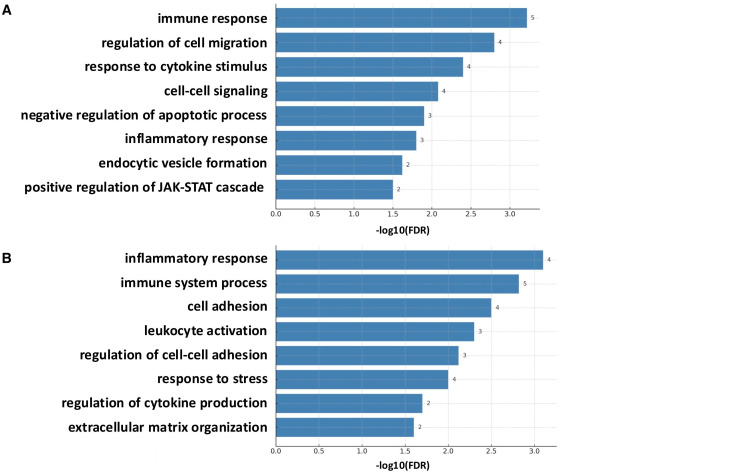
Pathway analysis of CLE EV proteins. (**A**) Pathway analysis of 18 proteins upregulated in CLE EVs compared to HCs and 4 CLE cohort specific proteins together. (**B**) Pathway analysis of 15 proteins downregulated in CLE EVs compared to HCs. Overrepresentation analysis was performed using GO Ontology terms, queried through the Python gprofiler-official package v1.0.0. The numbers next to the bar graphs indicate the number of associated proteins.

We analyzed the pathways involved in the 22 proteins that showed increased expression in CLE patients (18 proteins with increased expression and 4 proteins expressed only in CLE patients) and the 15 proteins that showed decreased expression. The former was found to be related to immune response, cell migration, cytokine response, and intercellular signaling (Figure 6A).

Among the proteins with decreased expression in CLE patients’ exosomes, some were related to inflammation, but the group with a decreased expression was characterized by the presence of numerous pathways related to adhesion molecules (Figure 6B).

## 4. Discussion

There have been significant advances in our understanding of the role of extracellular vesicles in disease over the last ten years. Various isolation and analysis methods are being developed. Since blood is highly rich in soluble proteins, it is important to control for protein contamination on EVs from blood. Size-exclusion chromatography (SEC) is a widely adopted method for the isolation of EVs and can efficiently remove highly abundant proteins [18]. We were able to collect highly pure EV-derived proteins by combining SEC with ultracentrifugation.

Previous studies have shown that EVs in the blood of SLE patients can induce inflammation and activate endothelial cells [19] but reports on the details of the EV proteins are limited. CD62P, a marker of platelet and endothelial cell activation, was upregulated in SLE patients and associated with SLEDAI scores [20]. Our bead-based flow cytometry results show that CD62P increased in the CLE group compared to the healthy controls (Figure 1A), whereas proteomic results show no difference between the two groups (Figure 1B). Differences between these two analyzes may indicate limitations in the sensitivity of proteomics analysis. Bead-based flow cytometry analysis covered major cell markers in blood, including neutrophils, T cells, B cells, and monocytes. The 10 of 11 surface markers that were increased on flow cytometry were also detected by proteomics analysis, and lest of markers were not detected. Based on the results of these two different analyses, it is believed that extracellular vesicles in blood are mainly derived from endothelial cells, platelets, and antigen-presenting cells.

Four proteins, SNRPB/SNRPN, OGN, IFI27, FBLN2, identified only in extracellular vesicles in the blood of CLE patients were also proteins reported to be related to lupus and inflammation. OGN is a gene that encodes mimecan, one of the proteoglycans. Mimecan is thought to be expressed in vascular smooth muscle cells (VSMCs), with increased expression during proliferation and aorta plaque formation [21]. Various studies have recently revealed that an increase in the amount of mimecan in serum is correlated with the onset of arteriosclerosis and cardiovascular disease [22,23]. Interferon alpha-inducible protein 27 (IFI27) is one of the interferon-stimulated genes (ISGs). Upregulation of the type I interferon signature is one of the most important features of systemic lupus erythematosus (SLE). IFI27 was reported as one of the six SLE-specific genes upregulated in SLE PBMCs relative to the healthy control [24]. Single-cell analysis using SLE PBMCs revealed that IFI27 was highly expressed in two types of monocytes, DCs, and plasma cells [25]. Fibulin-2 (FBLN2), an extracellular matrix (ECM) protein expressed in normal epithelia, is a kind of fibulin that is associated with basement membranes (BMs) and elastic ECM fibers. It is most highly expressed in normal skin and is important for the migration of keratinocytes and stabilization of the basement membrane during injury [26]. In the RNAseq analysis using formalin-fixed paraffin-embedded (FFPE) skin biopsies from juvenile dermatomyositis and CLE patients, Fibulin-2 was reported to be the most elevated JDM specific protein gene [27].

The 18 upregulated proteins found in the plasma EVs of CLE patients relative to the healthy controls included proteins associated with inflammation, lupus activity, and cardiovascular disease. Apolipoprotein E (APOE) [28,29] and alpha-1-antichymotrypsin (SERPINA3) [30,31] were both reported to positively correlate with cardiovascular events and lupus activity.

Galectin-3-binding protein (LGALS3BP) is highly expressed in lupus plasma EVs and is a lupus disease activity marker and a thrombus-related molecule [32].

Factor VII activating protease (FSAP), the official gene name is hyaluronic acid binding protein 2 (HABP2), is a circulating plasma serine protease. HABP2 has diverse effects on the regulation of apoptosis, inflammation, and coagulation [33].

NOTCH3 is overexpressed in the synovial fibroblasts of rheumatoid arthritis [34] patients and in the glomeruli of lupus nephritis [35]. The blockade of NOTCH3 signaling decreased inflammation in each mouse model. In rheumatoid arthritis, the mechanism involved a crosstalk between endothelial cells and fibroblasts. This crosstalk results in fibroblast transition from basal activity to an inflammatory program [29]. The presence of NOTCH3 in CLE patients’ plasma-derived EVs may suggest a similar crosstalk between a source tissue, possibly endothelial, and the skin. Transforming growth factor beta receptor type 3 (TGFBR3), one of the TGFβ receptors, was reported to be expressed in lupus nephropathy compared to other membranous nephropathies, although the positive rate was low [36]. Lysozyme (LYZ) is known as a bacteriolytic function associated with the monocyte-macrophage system and upregulated in SLE blood [37].

On the other hand, several proteins with anti-inflammatory effects also increased. Plxin-A4 is expressed on the vascular endothelium and negatively regulates inflammatory cell adhesion [38]. Serpin family A member 11 (SERPINA11) is not a well-understood protein, but it can inhibit cell growth, cell migration, and tumor metastasis in hepatocellular carcinoma patients [39].

As described above, many of the 18 proteins increased in CLE EVs correlate with pro-inflammatory effects and SLE. A number of proteins had an increased expression correlating with SLEDAI, while different proteins on EVs in CLE blood were associated with CLE pathology.

Interestingly, the 15 proteins whose expression was decreased in CLE EVs included multiple leukocyte adhesion molecules such as L-selectin (SELL), intercellular adhesion molecule 1 (ICAM1), CD43 (SPN), and tenascin (TNC, an integrin ligand). It is known that the type of integrin on cancer cell-derived EVs determines the organ into which the EV is taken up [40], and the inhibition of integrin reduces EV uptake into organs [41]. Therefore, it was expected that CLE disease-specific cell adhesion molecules would be elevated, but the results of this analysis show that multiple lymphocyte adhesion factors decreased. This supports the results of the pathway analysis indicating that many of the proteins that increased in CLE EVs are inflammation-related or cell migration-related, while many of the decreased proteins are lymphocyte adhesion proteins; however, because the number of detected proteins was limited, this interpretation should be made with caution. Interestingly, HABP2 and LYZ, both reported to be related to SLE, and ICAM1, a lymphocyte adhesion factor, were more correlated with the CLASI scores and protein expression than SLEDAI scores. Local inflammation in the skin may have systemic effects though EVs. Further investigation is necessary to determine the effects of these proteins on circulating EVs.

The present analysis is based on a small discovery cohort (CLE n = 6, HC n = 5), which limits statistical power and restricts the generalizability of the observed differences in adhesion molecules and pathway enrichment. These findings should be considered hypothesis-generating and validated in larger, independent cohorts, ideally with longitudinal sampling.

## 5. Conclusions

The types and amounts of proteins in plasma extracellular vesicles from CLE patients reflected systemic inflammation, but we were able to show that some proteins, such as LYZ, may also reflect cutaneous disease activity.

## Figures and Tables

**Table 1:** Clinical characteristics and disease activity measures of patients with cutaneous lupus erythematosus (CLE).

CLE No	Sex	Age	Type of CLE	CLASI	Treatments	Anti-Sm	SLEDAI	Components of SLEDAI	SLE
CLE 1	F	68	SCLE	33	HCQ, pred 20 mg, MMF1440 mg	+	2	2—new rash	Yes
CLE 2	F	74	DLE	9	MTX 15 mg	NA	4	2—new rash 2—alopecia	No
CLE 3	F	66	DLE/SCLE	35	HCQ	+	4	2—new rash, 2—alopecia	Yes
CLE 4	F	63	DLE	12	HCQ, MMF 1000 mg	−	4	2—new rash, 2—alopecia	No
CLE 5	F	65	SCLE	12	HCQ, MMF 1000 mg, lenalidamine	+	6	4—arthritis 2—new rash	Yes
CLE 6	F	33	DLE	29	HCQ, MMF 2000 mg, pred 10 mg	NA	12	4—proteinuria, 2—new rash, 2—mucosal ulcer, 2—low complement, 2—increased DNA binding	Yes

CLASI, Cutaneous Lupus Erythematosus Disease Area and Severity Index; CLE, cutaneous lupus erythematosus; DLE, discoid lupus erythematosus; F, female; HCQ, hydroxychloroquine; MMF, mycophenolate mofetil; MTX, methotrexate; NA, data not available; pred, prednisone; SCLE, subacute cutaneous lupus erythematosus; SLE, systemic lupus erythematosus; SLEDAI, Systemic Lupus Erythematosus Disease Activity Index; +, positive; −, negative.

## Data Availability

The data presented in this study are available on request from the corresponding author. The data are not publicly available due to ethical restrictions and patient privacy concerns.

## Supplementary Materials

The following supporting information can be downloaded at: https://www.mdpi.com/article/10.3390/cimb48010013/s1. References [15,16,42,43,44] are cited in the supplementary materials.
