# Supplementary material for: Extracellular Vesicular Proteins in Plasma from Patients with Cutaneous Lupus Correlate with Disease Activity
Source: Curr Issues Mol Biol. 2025 Dec 23;48(1):13. doi: 10.3390/cimb48010013 (PMC12840522; doi:10.3390/cimb48010013)
Supplement: Supplementary file 1 [file cimb-48-00013-s001.zip › cimb-3944802-supplementary.pdf]

Details of Proteomics Analysis

Protein extraction

Extracellular vesicles were solubilized in 50 µL of extraction buffer containing 5% sodium. dodecyl sulfate (SDS, Affymetrix), 50mM TEAB (pH 8.5, Sigma), and protease inhibitor cocktail (Roche cOmplete, EDTA free). To shear DNA and ensure complete solubilization, samples were sonicated for 10 minutes at 10°C in a Covaris R230 focused-ultrasonicator with the following settings: Dithering: Y=3.0, Speed=20.0, PIP: 360.0, DF: 30, CPB: 200. Samples were centrifuged at 3000g for 10 minutes to clarify lysate. 1 µL of each sample was taken to estimate protein concentration by in-gel staining with Bradford Coomassie solution and intensity analysis with GelAnalyzer 19.1, using a serial dilution of an in-house generated E.coli lysate standard.

In-solution digestion

100ug of each sample was digested using the S-Trap Micro (Protifi) per manufacturer's. protocol [1]. Briefly, proteins were reduced in 5mM TCEP (Thermo), alkylated in 20mM iodoacetamide (Sigma), and then acidified with phosphoric acid (Aldrich) to a final concentration of 1.2%. Samples were diluted with 90% methanol (Fisher) in 100 mM TEAB, loaded onto an S-trap column, and washed three times with 50/50 chloroform/methanol (Fisher) followed by three washes of 90% methanol in 100 mM TEAB. A 1:10 ratio (enzyme: protein) of Trypsin (Promega) and LysC (Wako) suspended in 20µL 50mM TEAB was added, and samples were digested for 1.5 hours at 47 °C in a humidity chamber. After incubation, peptides were eluted with an additional 40 µL of 50 mM TEAB, followed by 40 µL of 0.1% trifluoroacetic acid (TFA) (Pierce) in water, and finally 40 µL of 50/50 acetonitrile: water (Fisher) in 0.1% TFA. Eluates were combined and organic solvent was dried off via vacuum centrifugation. Samples were then desalted using an Oasis HLB µElution plate (30um, Waters). Wells were conditioned two times with 200 µL of acetonitrile and equilibrated three times with 200 µL of 0.1% TFA. Samples were applied, washed three times with 200 µL 0.1% TFA, and eluted directly into autosampler vials in three increments of 65 µL of 50:50 acetonitrile: water. Eluates were then dried by vacuum centrifugation and reconstituted in 0.1% TFA containing iRT peptides (Biognosys, Schlieren, Switzerland). Peptides were quantified with A280 measurement on a NanoDrop 1000 (Thermo) and adjusted to 0.4 ug/µL for injection.

Mass spectrometry data acquisition

Samples were randomized and analyzed on an Exploris 480 mass spectrometer (Thermofisher. Scientific San Jose, CA) coupled with an Ultimate 3000 nano UPLC system and an EasySpray source. Peptides were loaded onto an Acclaim PepMap 100 75um x 2cm trap column (Thermo) at 5uL/min, and separated by reverse phase (RP)-HPLC on a nanocapillary column, 75 µm id × 50cm 2um PepMap RSLC C18 column (Thermo). Mobile phase A consisted of 0.1% formic acid and mobile phase B of 0.1% formic acid/acetonitrile. Peptides were eluted into the mass spectrometer at 300 nL/min with each RP-LC run comprising a 105 minute gradient from 3% B to 45% B.

Data independent acquisition (DIA) mass spectrometer settings were as follows: one 120,000 resolution full MS scan with a scan range of 350-1200 m/z, normalized automatic gain control (AGC) target of 300%, and automatic maximum inject time. This was followed by variable (DIA) isolation windows for the MS2 scans at 30,000 resolution, normalized AGC target of 1000%, and automatic injection time. The default charge state was 3, the first mass was fixed at 250 m/z, and the normalized collision energy for each window was set at 27.

QA/QC and system suitability

The suitability of Exploris 480 instrument was monitored using QuiC software (Biognosys; Schlieren, Switzerland) for the analysis of the spiked-in iRT peptides. As a measure for quality control, we injected standard E. coli protein digest in between samples (one injection after every four biological samples) and collected the data in data dependent acquisition (DDA) mode. The collected DDA data

were analyzed in MaxQuant [3] and the output was subsequently visualized using the PTXQC [4] package to track the quality of the instrumentation.

Mass spectrometry raw data processing

The raw files for DIA analysis were processed with Spectronaut [2] version 16 in Direct-DIA mode using reference human proteome from UniProt (42,247 reviewed canonical and isoform proteins). The default settings in Spectronaut were used for peptide and protein quantification with cysteine carbamidomethylation as fixed modification and methionine oxidation and protein N-terminal Acetylation as variable modifications. The protein MS2 intensity values were measured and cross-run normalized by Spectronaut. Data were filtered out at a false discovery rate (FDR) of 1% at precursor, peptide and protein level.

Bioinformatics analysis

Data processing and statistical analysis was performed in Perseus 1.6.14.0 [5]. The raw MS2 intensity data were log2 transformed and normalized by subtracting the median for each sample. After data filtering, t-test was employed to identify differentially abundant proteins between high and no-activity fractions using P.Value < 0.05 as significant threshold. The proteins that were exclusively detected in one experimental group were also reported for further bioinformatics analysis.

Supplymental References

1. Zougman, A.; Selby, P.J.; Banks, R.E. Suspension trapping (STrap) sample preparation method for bottom-up proteomics analysis. *Proteomics* **2014**, *14,* 1006–1000. doi: 10.1002/pmic.201300553. PMID: 24678027.

2. Bruderer, R.; Bernhardt, O.M.; Gandhi, T.; Miladinović, S.M.; Cheng, L.Y.; Messner, S.; Ehrenberger, T.; Zanotelli, V.; Butscheid, Y.; Escher, C.; et al. Extending the limits of quantitative proteome profiling with data-independent acquisition and application to acetaminophen-treated three-dimensional liver microtissues. *Mol. Cell Proteom.* **2015**, *14,* 1400–1410. doi:10.1074/mcp.M114.044305. PMID: 25724911; PMCID: PMC4424408.

3. Tyanova, S.; Temu, T.; Cox, J. The MaxQuant computational platform for mass spectrometry-based shotgun proteomics. *Nat. Protoc*. **2016**, *11*, 1301-2319. https://doi.org/10.1038/nprot.2016.136. PMID: 27809316.

4. Bielow, C.; Mastrobuoni, G.; Kempa, S. Proteomics Quality Control: Quality Control Software for. MaxQuant Results. *J. Proteome Res.* **2016**, *15*, 777–787. https://doi.org/10.1021/acs.jproteome.5b00780. PMID: 26653327.

5. Tyanova, S.; Temu, T.; Sinitcyn, P.; Carlson, A.; Hein, M.Y.; Geiger, T.; Mann, M.; Cox, J. The Perseus. computational platform for comprehensive analysis of (prote)omics data. *Nat Methods* **2016**, *13*, 731–740. https://doi.org/10.1038/nmeth.3901. PMID: 27348712.
