# Supplementary material for: Extracellular Vesicular Proteins in Plasma from Patients with Cutaneous Lupus Correlate with Disease Activity
Source: Curr Issues Mol Biol. 2025 Dec 23;48(1):13. doi: 10.3390/cimb48010013 (PMC12840522; doi:10.3390/cimb48010013)
Supplement: Supplementary file 1 [file cimb-48-00013-s001.zip › Supplemental Figure S1.pptx]

## Slide 1
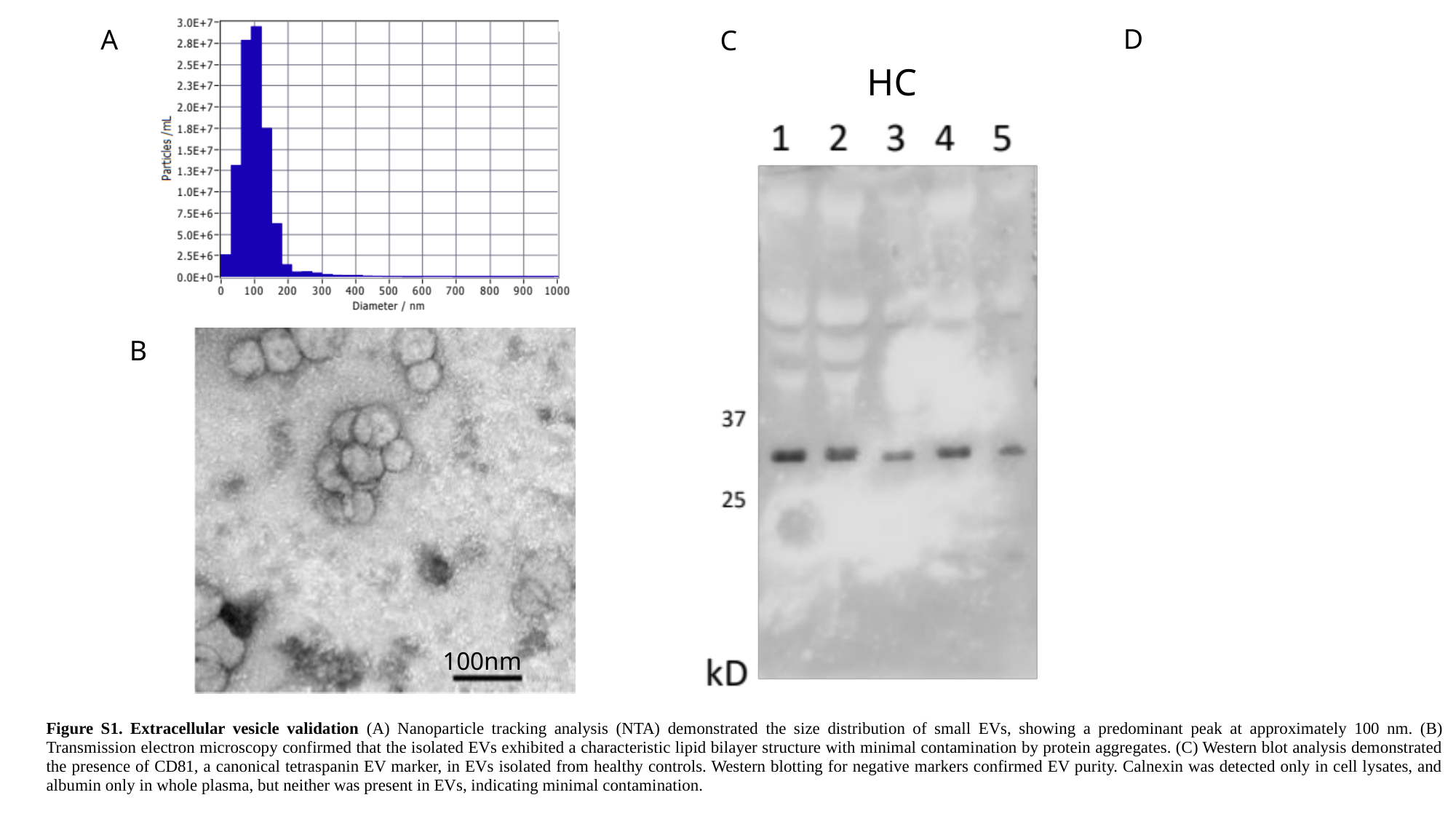

D
A
C
HC
B
100nm
Figure S1. Extracellular vesicle validation (A) Nanoparticle tracking analysis (NTA) demonstrated the size distribution of small EVs, showing a predominant peak at approximately 100 nm. (B) Transmission electron microscopy confirmed that the isolated EVs exhibited a characteristic lipid bilayer structure with minimal contamination by protein aggregates. (C) Western blot analysis demonstrated the presence of CD81, a canonical tetraspanin EV marker, in EVs isolated from healthy controls. Western blotting for negative markers confirmed EV purity. Calnexin was detected only in cell lysates, and albumin only in whole plasma, but neither was present in EVs, indicating minimal contamination.
