# Supplementary material for: Extracellular Vesicular Proteins in Plasma from Patients with Cutaneous Lupus Correlate with Disease Activity
Source: Curr Issues Mol Biol. 2025 Dec 23;48(1):13. doi: 10.3390/cimb48010013 (PMC12840522; doi:10.3390/cimb48010013)
Supplement: Supplementary file 1 [file cimb-48-00013-s001.zip › supplementary Table S1,2.pdf]

UniProt ID	Genes	p-value
P61626	LYZ	2.72
Q08380	LGALS3BP	2.29
A0A0C4DH33	IGHV1-24	2.28
Q8WWA1	TMEM40	2.27
Q86U17	SERPINA11	2.26
A0A0C4DH32	IGHV3-20	1.99
P01011	SERPINA3	1.95
P58166	INHBE	1.94
Q93050	ATP6V0A1	1.94
P02649	APOE	1.87
P51452	DUSP3	1.84
Q14520	HABP2	1.73
Q9UHG3	PCYOX1	1.72
Q9UM47	NOTCH3	1.66
Q9NP58	ABCB6	1.66
Q9HCM2	PLXNA4	1.66
P19397	CD53	1.65
P14151	SELL	1.65
P61006	RAB8A	1.64
P99999	CYCS	1.64
Q15208	STK38	1.63
P04844	RPN2	1.57
P00736	C1R	1.54
P05362	ICAM1	1.52
P33908	MAN1A1	1.45
Q03167	TGFBR3	1.41
A0A087WSX0	IGLV5-45	1.36
A0A0C4DH29	IGHV1-3	1.35
P16150	SPN	1.35
Q02818	NUCB1	1.34
P24821	TNC	1.34
O00560	SDCBP	1.34
P22748	CA4	1.34

Supplementary Table S1

Supplementary Table S2. Clinical characteristics of healthy controls (HCs)

HC No	Sex	Age	Race
HC 1	M	26	Caucasian
HC 2	F	35	Asian
HC 3	F	22	African American
HC 4	M	63	Caucasian
HC 5	F	54	Caucasian

Inclusion criterion: Individuals without any diagnosed disease at the time of blood collection.
